# Supplementary material for: Wakefulness Induced by TAAR1 Partial Agonism in Mice Is Mediated Through Dopaminergic Neurotransmission
Source: Int J Mol Sci. 2024 Oct 22;25(21):11351. doi: 10.3390/ijms252111351 (PMC11547084; doi:10.3390/ijms252111351)
Supplement: Supplementary file 1 [file ijms-25-11351-s001.zip › ijms-3220377-supplementary.pdf]

SUPPLEMENTARY INFORMATION

"Wakefulness Induced by TAAR1 Partial Agonism is Mediated Through Dopaminergic Neurotransmission"

Sunmee Park[1], Jasmine Heu[1], Marius C. Hoener[2], Thomas S. Kilduff[1]

[1]Center for Neuroscience, Biosciences Division, SRI International, Menlo Park, CA

[2]Neuroscience, Ophthalmology and Rare Diseases DTA, pRED, Roche Innovation Center Basel, F. Hoffmann-La Roche Ltd, Basel, Switzerland

This file includes:

Figures S1 to S5
Tables S1 to S2

Supplementary Figures

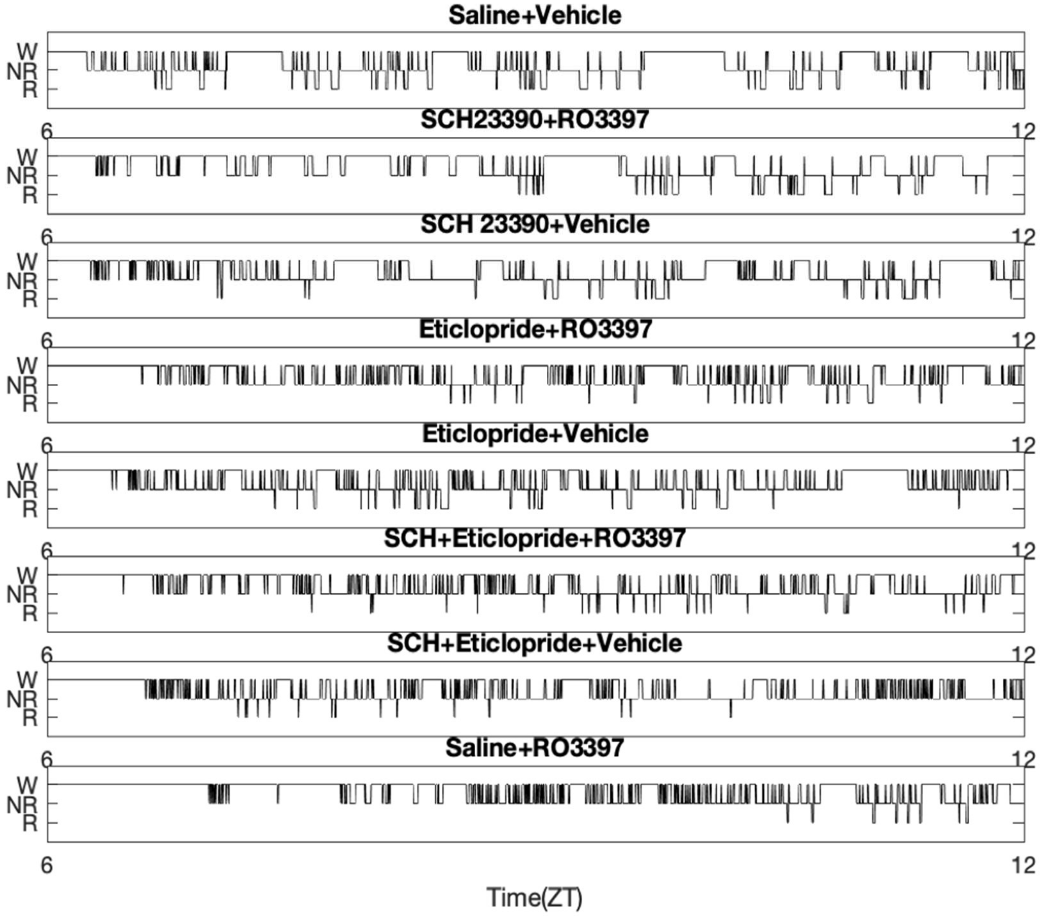

Figure S1. Example hypnograms of each treatment condition. Hypnograms of male C57BL6/J mice recorded from ZT6 to ZT12. Each hypnogram represents one mouse from each treatment group, illustrating the variability in distribution of sleep/wake states (Wake, NREM, and REM) across the 6-hour recording period after dosing at ZT6.

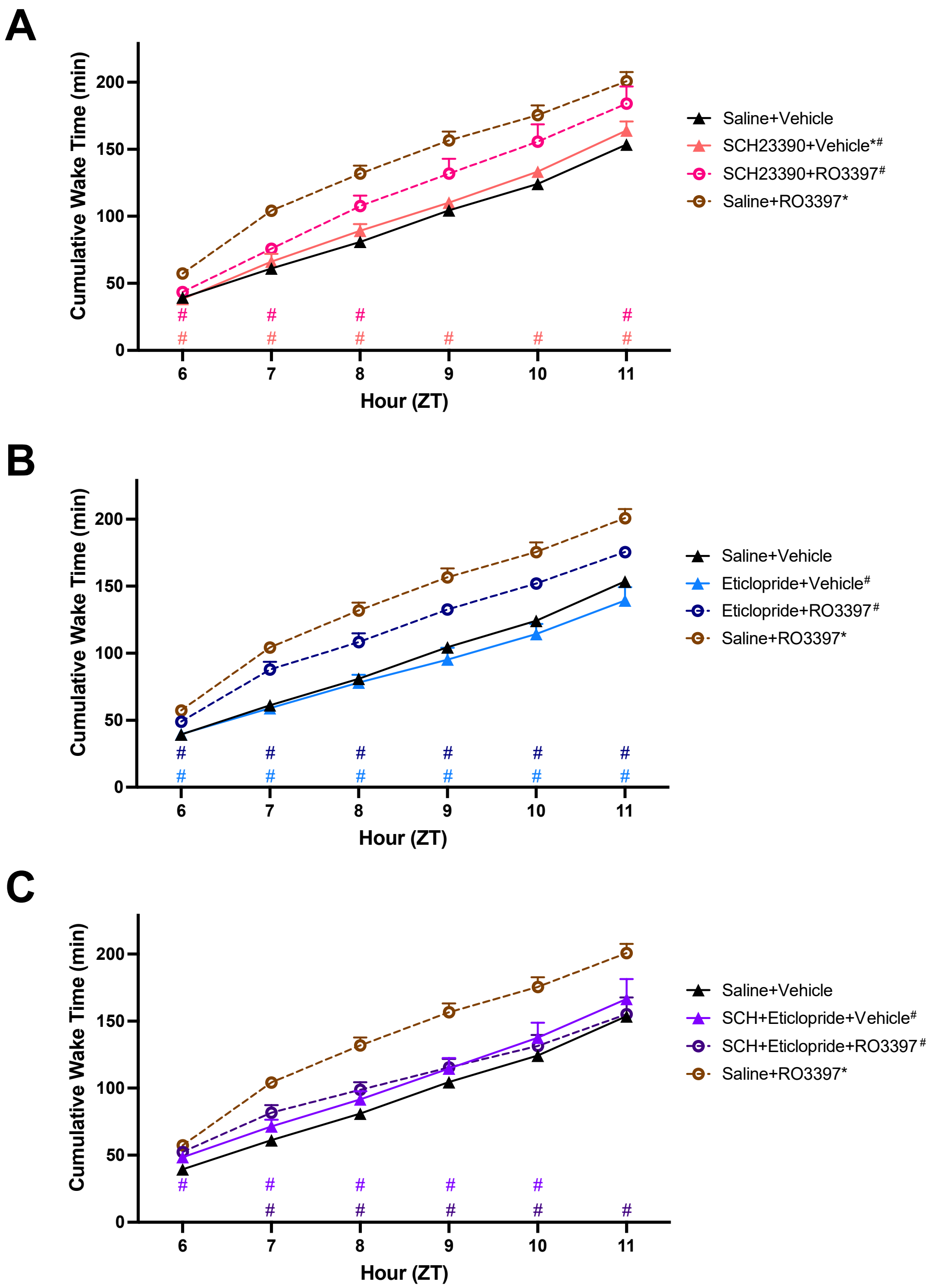

Figure S2. Cumulative Wake time for the first 6 hours after the second dosing. For ease of visualization, data are split into three subgroups in which the results from the negative (Sal+Veh) and positive (Sal+RO3397) control treatments are repeated in each graph. **A-C**. Cumulative Wake time (mean+SEM). Colored symbols indicate statistical significance for that hour compared to Sal+Veh(*) or Sal+RO3397(#) based on RM-ANOVA. *, # $p < 0.05$;

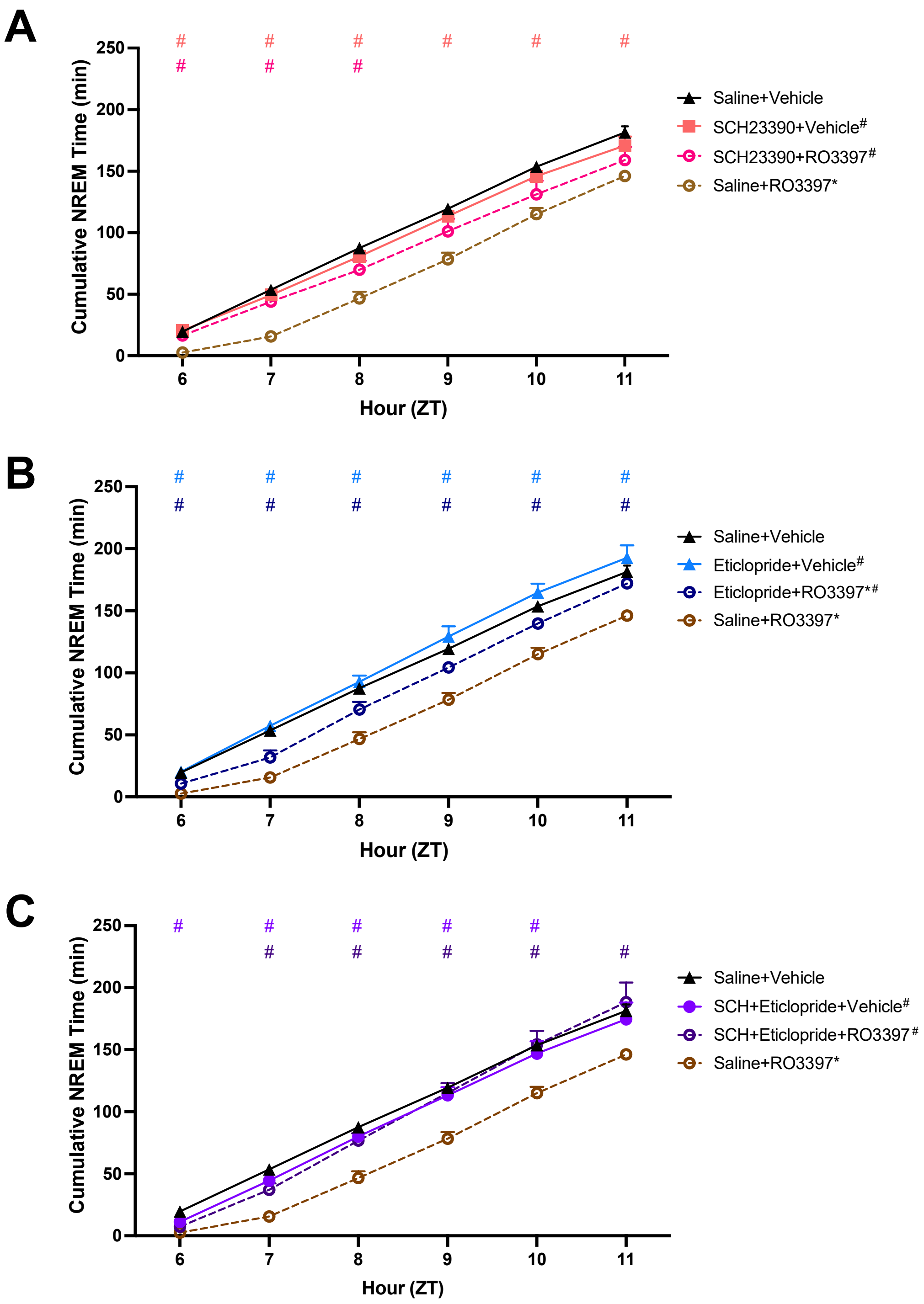

Figure S3. Cumulative NREM time for the first 6 hours after the second dosing. For ease of visualization, data are split into three subgroups in which the results from the negative (Sal+Veh) and positive (Sal+RO3397) control treatments are repeated in each graph. **A-C**. Cumulative NREM time (mean+SEM). Colored symbols indicate statistical significance for that hour compared to Sal+Veh(*) or Sal+RO3397(#) based on RM-ANOVA. *, # $p < 0.05$;

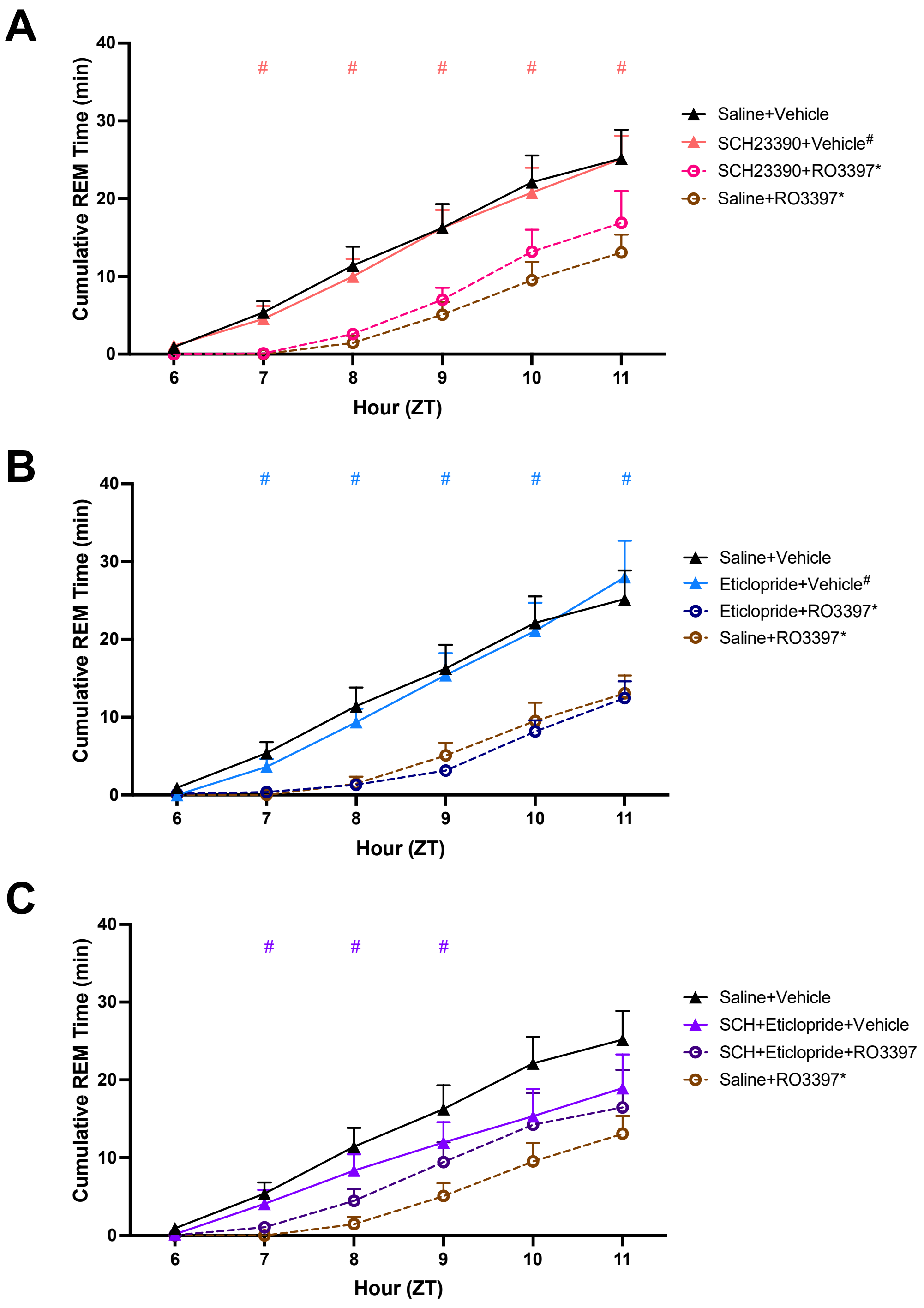

Figure S4. Cumulative REM time for the first 6 hours after the second dosing. For ease of visualization, data are split into three subgroups in which the results from the negative (Sal+Veh) and positive (Sal+RO3397) control treatments are repeated in each graph. **A-C**. Cumulative REM time (mean+SEM). Colored symbols indicate statistical significance for that hour compared to Sal+Veh(*) or Sal+RO3397(#) based on RM-ANOVA. *, # $p < 0.05$;

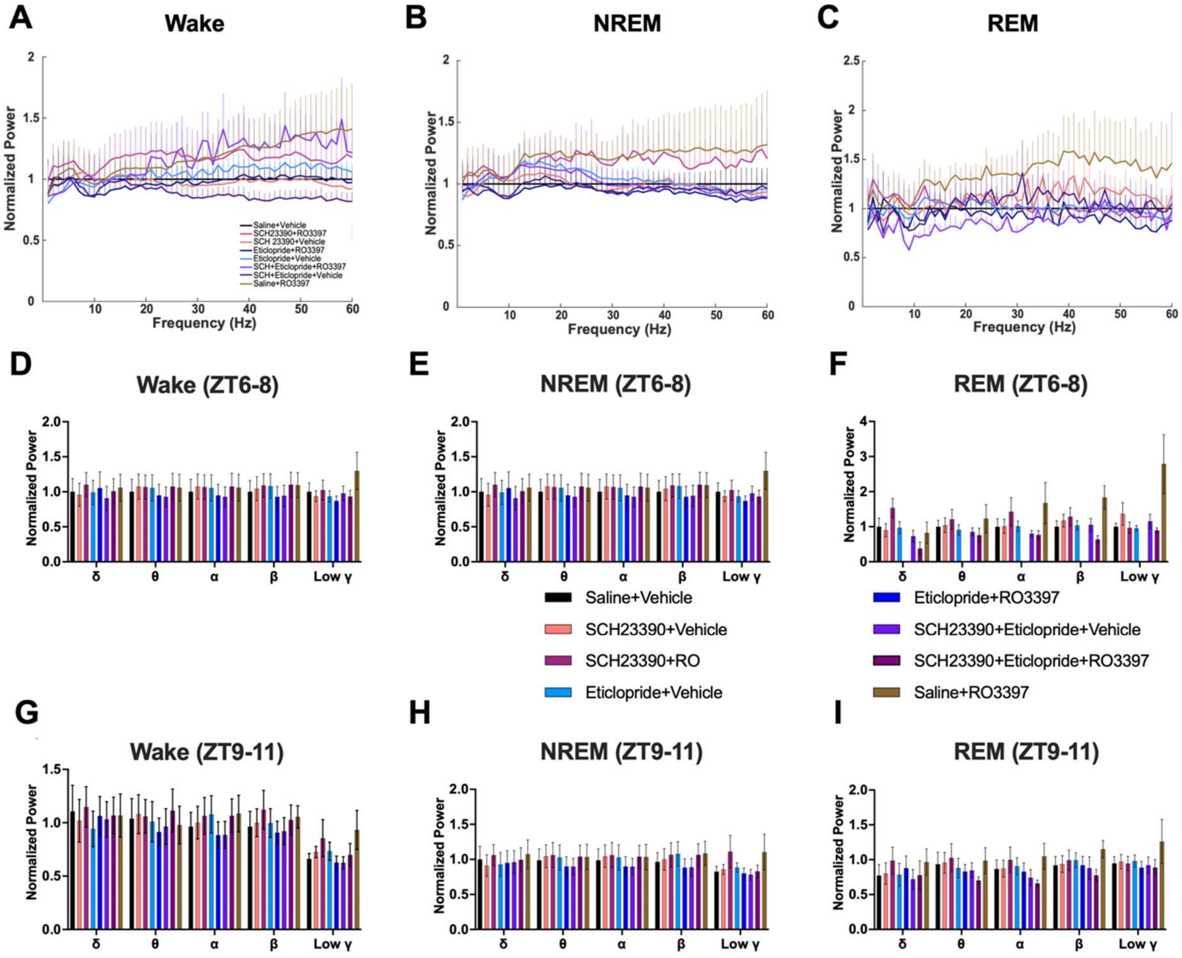

Figure S5. EEG spectral power (0-60 Hz) for each treatment during the first 6 post-dosing hours. A-C. 6-hour average spectral power for (A) Wake, (B) NREM sleep and (C) REM sleep. **D-F**. Normalized EEG power binned into the conventional bandwidths for each treatment during the first 3 post-dosing hours (ZT6-8) for (D) Wake, (E) NREM sleep and (F) REM sleep. **G-I**. Normalized EEG power binned into the conventional bandwidths for each treatment during the second 3 post-dosing hours (ZT9-11) for (G) Wake (H) NREM sleep and (I) REM sleep.

Table S1. Statistics underlying Figures and Supplementary Figures.

Figure/Panel (Time bin)	Data Structure	Type of test	*F* value	*p* value	Variable
**1B-D**	normal distribution	2-way ANOVA (Condition)	3.919	0.0019	ZT6-ZT11 hourly wake time (min)
1B	normal distribution	Tukey's multiple comparison		<0.0001	Saline+Vehicle vs. Saline+RO3397
1C	normal distribution	Tukey's multiple comparison		0.0060	Eticlopride+RO3397 vs. Saline+RO3397
1D	normal distribution	Tukey's multiple comparison		0.0075	SCH23390+eticlopride+RO3397 vs. Saline+RO3397
**1E-1G**	normal distribution	2-way ANOVA (Condition x Time)	5.235	0.0002	ZT6-8. ZT9-11 3-hour bin total wake time (min)
1E,1F,1G	normal distribution	Tukey's multiple comparison		<0.0001	Saline+Vehicle vs. Saline+RO3397
1E	normal distribution	Tukey's multiple comparison		0.0002	SCH23390+Vehicle vs. Saline+RO3397
1F	normal distribution	Tukey's multiple comparison		<0.0001	Eticlopride+Vehicle vs. Saline+RO3397
1G	normal distribution	Tukey's multiple comparison		0.0005	SCH23390+eticlopride+Vehicle vs. Saline+RO3397
1G	normal distribution	Tukey's multiple comparison		0.0086	SCH23390+eticlopride+RO3397 vs. Saline+RO3397
**2A-C**	heterogeneous dist	Brown-Forsythe ANOVA test	15.990	<0.0001	ZT6-12 NREM latency
		Welch's ANOVA	12.850	<0.001	ZT6-12 NREM latency
2A,B,C	normal distribution	Dunnett's multiple comparison		0.0005	Saline+Vehicle vs. Saline+RO3397
2A	normal distribution	Dunnett's multiple comparison		0.0003	SCH23390+Vehicle vs. Saline+RO3397
2A	normal distribution	Dunnett's multiple comparison		0.0004	SCH23389+RO3397 vs. Saline+RO3397
2B	normal distribution	Dunnett's multiple comparison		0.0003	Eticlopride+Vehicle vs. Saline+RO3397
2C	normal distribution	Dunnett's multiple comparison		0.0076	SCH23390+eticlopride+Vehicle vs. Saline+RO3397
**2D-F**	normal distribution	2-way ANOVA (Condition)	2.391	0.0350	ZT6-NT12 hourly NREM time (min)
2D,E,F	normal distribution	Tukey's multiple comparison		0.0003	Saline+Vehicle vs. Saline+RO3397
2D	normal distribution	Tukey's multiple comparison		0.0164	SCH23390+Vehicle vs. Saline+RO3397
2E	normal distribution	Tukey's multiple comparison		0.0013	Eticlopride+Vehicle vs. Saline+RO3397
2E	normal distribution	Tukey's multiple comparison		0.0023	Eticlopride+RO3397 vs. Saline+RO3397
2F	normal distribution	Tukey's multiple comparison		0.0256	SCH23390+eticlopride+RO3397 vs. Saline+RO3397
**2G-I**	normal distribution	2-way ANOVA (Condition x Time)	4.179	0.0012	ZT6-9, ZT9-12 3-hour bin total NREM time (min)
2G,2H,2I	normal distribution	Tukey's multiple comparison		0.0004	Saline+Vehicle vs. Saline+RO3397
2G	normal distribution	Tukey's multiple comparison		0.0060	SCH23390+Vehicle vs. Saline+RO3397
2H	normal distribution	Tukey's multiple comparison		<0.0001	Eticlopride+Vehicle vs. Saline+RO3397
2I	normal distribution	Tukey's multiple comparison		0.0078	SCH23390+eticlopride+Vehicle vs. Saline+RO3397
2I	normal distribution	Tukey's multiple comparison		0.0246	SCH23390+eticlopride+RO3397 vs. Saline+RO3397
**3A-C**	heterogeneous dist	Brown-Forsythe ANOVA test	10.550	<0.0001	ZT6-12 REM latency
		Welch's ANOVA	8.901	<0.0001	ZT6-12 REM latency
3A,B,C	normal distribution	Dunnett's multiple comparison		0.0019	Saline+Vehicle vs. Saline+RO3397
3A	normal distribution	Dunnett's multiple comparison		0.0013	SCH23390+Vehicle vs. Saline+RO3397
3B	normal distribution	Dunnett's multiple comparison		0.0106	Eticlopride+Vehicle vs. Saline+RO3397
3C	normal distribution	Dunnett's multiple comparison		0.0466	SCH23390+eticlopride+Vehicle vs. Saline+Vehicle
3C	normal distribution	Dunnett's multiple comparison		0.0233	SCH23390+eticlopride+Vehicle vs. Saline+RO3397
**3D-F**	normal distribution	2-way ANOVA (Condition x Time)	2.458	0.0307	NT6-NT12 NREM hourly time (min)
3D,E,F	normal distribution	Tukey's multiple comparison		0.0167	Saline+Vehicle vs. Saline+RO3397
3D	normal distribution	Tukey's multiple comparison		0.0073	SCH23390+Vehicle vs. Saline+RO3397
3E	normal distribution	Tukey's multiple comparison		0.0146	Eticlopride+Vehicle vs. Saline+RO3397
**3G-I**	normal distribution	2-way ANOVA (Condition x Time)	2.473	0.0299	ZT6-9, ZT9-12 3-hour bin total NREM time (min)
3G,H,I	normal distribution	Tukey's multiple comparison		0.0470	Saline+Vehicle vs. Saline+RO3397
**4A-C**	normal distribution	2-way ANOVA (Condition x Time)	2.490	0.0290	ZT6-ZT12 Raw Temperature
4B	normal distribution	Tukey's multiple comparison		0.0170	Eticlopride+Vehicle vs. Saline+Vehicle
4C	normal distribution	Tukey's multiple comparison		0.0130	SCH23390+eticlopride+Vehicle vs. Saline+RO3397
4C	normal distribution	Tukey's multiple comparison		0.0320	SCH23390+eticlopride+RO3397 vs. Saline+RO3397
**4D-F**	normal distribution	2-way ANOVA (Condition x Time)	9.660	<0.0001	ZT6-ZT12 LMA (counts/min)
4D,E,F	normal distribution	Tukey's multiple comparison		0.0467	Saline+Vehicle vs. Saline+RO3397
4E	normal distribution	Tukey's multiple comparison		0.0001	Eticlopride+Vehicle vs. Saline+Vehicle
4E	normal distribution	Tukey's multiple comparison		0.0004	Eticlopride+RO3397 vs. Saline+Vheicle
4E	normal distribution	Tukey's multiple comparison		0.0014	Eticlopride+Vehicle vs. Saline+RO3397
4E	normal distribution	Tukey's multiple comparison		0.0061	Eticlopride+RO3397 vs. Saline+RO3397
4F	normal distribution	Tukey's multiple comparison		0.0002	SCH23390+eticlopride+Vehicle vs. Saline+Vehicle
4F	normal distribution	Tukey's multiple comparison		0.0001	SCH23390+eticlopride+RO3397 vs. Saline+Vehicle

Figure/Panel (Time bin)	Data Structure	Type of test	*F* value	*p* value	Variable
4F	normal distribution	Tukey's multiple comparison		0.0001	SCH23390+eticlopride+RO3397 vs. Saline+Vehicle
4F	normal distribution	Tukey's multiple comparison		0.0030	SCH23390+eticlopride+Vehicle vs. Saline+RO3397
4F	normal distribution	Tukey's multiple comparison		0.0013	SCH23390+eticlopride+RO3397 vs. Saline+RO3397
**Fig.S1**	normal distribution	2-way ANOVA (Condition x Time)	2.882	<0.0001	ZT6-11 Cumulative Wake Time
Fig.S1A,B,C	normal distribution	Tukey's multiple comparison		<0.0001	Saline+Vehicle vs. Saline+RO3397
Fig.S1A	normal distribution	Tukey's multiple comparison		<0.0001	SCH23390+Vehicle vs. Saline+RO3397
Fig.S1A	normal distribution	Tukey's multiple comparison		0.0464	SCH23390+RO3397 vs. Saline+RO3397
Fig.S1B	normal distribution	Tukey's multiple comparison		<0.0001	Eticopride+Vehicle vs. Saline+RO3397
Fig.S1B	normal distribution	Tukey's multiple comparison		0.0033	Eticlopride+RO3397 vs. Saline+RO3397
Fig.S1C	normal distribution	Tukey's multiple comparison		0.0025	SCH23390+eticlopride+Vehicle vs. Saline+RO3397
Fig.S1C	normal distribution	Tukey's multiple comparison		0.0016	SCH23390+eticlopride+RO3397 vs. Saline+RO3397
**Fig.S2**	normal distribution	2-way ANOVA (Condition x Time)	5.474	0.0010	ZT6-11 Cumulative NREM Time
Fig.S2A	normal distribution	2-way ANOVA (Condition x Time)		0.0001	SCH23390+Vehicle vs. Saline+RO3397
Fig.S2A	normal distribution	Tukey's multiple comparison		0.0401	SCH23390+RO3397 vs. Saline+RO3397
Fig.S2B	normal distribution	Tukey's multiple comparison		0.0001	Eticopride+Vehicle vs. Saline+RO3397
Fig.S2B	normal distribution	Tukey's multiple comparison		0.0007	Eticlopride+RO3397 vs. Saline+RO3397
Fig.S2C	normal distribution	Tukey's multiple comparison		0.0025	SCH23390+eticlopride+Vehicle vs. Saline+RO3397
Fig.S2C	normal distribution	Tukey's multiple comparison		0.0050	SCH23390+eticlopride+RO3397 vs. Saline+RO3397
3H (ZT19-ZT24)	normal distribution	2-way ANOVA (Condition x Time)	4.761	<0.0001	ZT19-ZT24 Normalized NR Beta Power
3H (ZT1-ZT6)	normal distribution	2-way ANOVA (Condition x Time)	2.543	0.0008	ZT1-ZT6 Normalized NR Beta Power
3H (ZT7-ZT12)	normal distribution	2-way ANOVA (Condition x Time)	1.589	0.0632	ZT7-ZT12 Normalized NR Beta Power
4A (ZT19-ZT24)	normal distribution	2-way ANOVA (Condition x Time)	2.660	0.0027	WT mice Cumulative W Time (min)
4B (ZT19-ZT24)	normal distribution	2-way ANOVA (Condition x Time)	2.780	0.0003	HET mice Cumulative W Time (min)
4C (ZT19-ZT24)	normal distribution	2-way ANOVA (Condition x Time)	1.040	0.4274	KO mice Cumulative W Time (min)
4D (ZT19-ZT24)	normal distribution	2-way ANOVA (Condition x Time)	1.980	0.0266	WT mice Cumulative NR Time (min)
4E (ZT19-ZT24)	normal distribution	2-way ANOVA (Condition x Time)	2.870	0.0002	HET mice Cumulative NR Time (min)
4F (ZT19-ZT24)	normal distribution	2-way ANOVA (Condition x Time)	1.010	0.4548	KO mice Cumulative NR Time (min)

Table S2. Basal sleep/wake parameters of male C57BL6/J mice treated with a dopaminergic antagonist followed by TAAR1 agonist or vehicle.

		Time (min)		Number of bouts		Mean Bout Duration (min)	
Treatment Condition	N	ZT6-8	ZT9-11	ZT6-8	ZT9-11	ZT6-8	ZT9-11
WAKE							
Saline+Vehicle	7	81.0 ± 3.4	72.5 ± 2.6	28.4 ± 3.0	29.0 ± 3.0	3.0 ± 0.5	2.6 ± 0.3
SCH23390+Vehicle	7	$89.2 \pm 5.0^{\dagger\dagger\dagger}$	74.9 ± 5.6	36.0 ± 2.8	25.6 ± 1.5	2.4 ± 0.2	2.9 ± 0.3
SCH23390+RO3397	7	107.6 ± 7.8	76.5 ± 7.2	37.0 ± 3.3	$31.3 \pm 4.8^{\dagger\dagger\dagger\dagger}$	2.9 ± 0.3	2.6 ± 0.4
Eticlopride+Vehicle	7	$78.0 \pm 5.9^{\dagger\dagger\dagger\dagger}$	61.3 ± 5.4	$52.9 \pm 4.2^{**}$	$46.7 \pm 4.6^{\dagger\dagger\dagger\dagger}$	$1.4 \pm 0.2^{\dagger\dagger\dagger\dagger}$	1.3 ± 0.1
Eticlopride+RO3397	7	108.3 ± 6.6	67.2 ± 7.6	$56.4 \pm 5.8^{***}$	$45.7 \pm 5.1^{\dagger\dagger\dagger\dagger}$	$2.0 \pm 0.3^{\dagger\dagger\dagger\dagger}$	1.6 ± 0.4
SCH23390+Eticlopride+Vehicle	7	$91.6 \pm 6.1^{\dagger\dagger\dagger}$	74.9 ± 10.4	$57.3 \pm 8.7^{***}$	$52.71 \pm 4.14^{\dagger\dagger\dagger\dagger}$	$1.8 \pm 0.3^{\dagger\dagger\dagger\dagger}$	1.4 ± 0.2
SCH23390+Eticlopride+RO3397	7	$98.8 \pm 5.7^{\dagger\dagger}$	56.4 ± 8.4	$57.0 \pm 4.8^{***}$	$45.9 \pm 6.3^{\dagger\dagger\dagger\dagger}$	$1.8 \pm 0.2^{\dagger\dagger\dagger\dagger}$	1.2 ± 0.1
Saline+RO3397	7	$131.9 \pm 5.9^{****}$	68.9 ± 3.1	$33.0 \pm 3.6^{**}$	35.3 ± 5.7	4.3 ± 0.6	2.1 ± 0.3
2-way ANOVA		$F_{(7,48)} = 3.919$; $p = 0.002$		$F_{(7,48)} = 21.19$; $p < 0.0001$		$F_{(7,48)} = 9.56$; $p < 0.0001$	
NREM Sleep							
Saline+Vehicle	7	87.6 ± 2.5	93.8 ± 3.1	48.9 ± 4.6	49.0 ± 6.0	1.9 ± 0.3	2.1 ± 0.3
SCH23390+Vehicle	7	$80.8 \pm 3.8^{\dagger\dagger}$	90.0 ± 6.1	53.1 ± 4.1	43.6 ± 3.1	1.6 ± 0.2	2.1 ± 0.1
SCH23390+RO3397	7	69.8 ± 7.3	89.2 ± 4.6	47.6 ± 4.0	48.1 ± 5.3	1.5 ± 0.2	2.0 ± 0.3
Eticlopride+Vehicle	7	$92.6 \pm 5.2^{\dagger\dagger\dagger\dagger}$	100.1± 6.4	$72.7 \pm 5.3^{*\dagger\dagger\dagger\dagger}$	68.4 ± 3.8	1.3 ± 0.1	1.5 ± 0.1
Eticlopride+RO3397	7	70.4 ± 6.1	101.7 ± 8.6	$65.7 \pm 3.9^{\dagger\dagger\dagger}$	67.4 ± 5.2	$1.0 \pm 0.1^{*}$	1.5 ± 0.1
SCH23390+Eticlopride+Vehicle	7	$80.1 \pm 5.0^{\dagger\dagger}$	94.5 ± 10.2	$71.9 \pm 6.1^{*\dagger\dagger\dagger\dagger}$	72.7 ± 6.0*	1.1 ± 0.1	1.3 ± 0.2
SCH23390+Eticlopride+RO3397	7	$76.8 \pm 5.9^{\dagger}$	111.6 ± 11.02	$61.7 \pm 7.6^{\dagger\dagger}$	68.4 ± 5.6	1.3 ± 0.2	1.7 ± 0.3
Saline+RO3397	7	$46.7 \pm 5.3^{***}$	99.5 ± 3.0	32.4 ± 3.3	55.7± 5.5	1.4 ± 0.1	1.9 ± 0.2
2-way ANOVA		$F_{(7,48)} = 2.391$; $p = 0.035$		$F_{(7,48)} = 8.347$; $p < 0.0001$		$F_{(7,48)} = 2.50$; $p = 0.0283$	
REM Sleep							
Saline+Vehicle	7	11.4 ± 2.4	13.8 ± 2.1	12.0 ± 3.3	15.1 ± 2.2	1.0 ± 0.2	0.8 ± 0.1
SCH23390+Vehicle	7	10.0± 2.2	15.1 ± 1.2	9.0 ± 2.1	14.6 ± 1.6	1.0 ± 0.1	1.0 ± 0.1
SCH23390+RO3397	7	2.6 ± 0.7	14.3 ± 3.9	2.6 ± 0.7	14.1 ± 4.2	1.1 ± 0.3	1.0 ± 0.1
Eticlopride+Vehicle	7	9.4± 1.7	18.6 ± 3.2	11.4 ± 2.1	24.6 ± 4.5	0.8 ± 0.1	0.8 ± 0.1
Eticlopride+RO3397	7	1.3 ± 0.5*	11.1 ± 1.9	1.7 ± 0.7	17.9 ± 2.4	$0.4 \pm 0.0^{*}$	0.5 ± 0.1
SCH23390+Eticlopride+Vehicle	7	8.4 ± 2.1	10.6 ± 3.2	15.4 ± 3.6	18.4 ± 5.9	$0.4 \pm 0.0^{*}$	0.5 ± 0.1
SCH23390+Eticlopride+RO3397	7	4.5 ± 1.5	12.0 ± 3.6	7.3 ± 2.6	16.7 ± 5.4	$0.4 \pm 0.0^{*}$	0.6 ± 0.1
Saline+RO3397	7	1.4 ± 0.9*	11.6 ± 1.5	1.9 ± 0.8	12.9 ± 1.5	0.6 ± 0.2	0.86 ± 0.07
2-way ANOVA		$F_{(7,48)} = 2.473$; $p = 0.030$		$F_{(7,48)} = 2.204$; $p = 0.050$		$F_{(7,48)} = 7.80$; $p < 0.0001$	

*p < 0.05; **p < 0.01; ***p < 0.001; ****p < 0.0001 vs. Saline+Vehicle

†p < 0.05; ††p < 0.01; †††*p* < 0.001; ††††*p* < 0.0001 vs. Saline+RO3397
